# Evolution of Technique in Endoscopic Transsphenoidal Surgery for Pituitary Adenoma: A Single Institution Experience from 220 Procedures

**DOI:** 10.7759/cureus.2010

**Published:** 2018-01-01

**Authors:** Ake Hansasuta, Siriwut Pokanan, Pritsana Punyawai, Wattana Mahattanakul

**Affiliations:** 1 Division of Neurosurgery, Department of Surgery, Faculty of Medicine Ramathibodi Hospital, Mahidol University

**Keywords:** endoscopic, endonasal, transsphenoidal, pituitary adenoma, minimally invasive, learning curve, advanced instrument, tools

## Abstract

Introduction

Endoscopic transsphenoidal surgery (ETSS) for pituitary adenoma (PA) has been a recent shift from the traditional microscopic technique. Although some literature demonstrated superiority of ETSS over the microscopic method and some evaluated mono- vs. binostril access within the ETSS, none had explored the potential influence of dedicated instrument, as this procedure had evolved, on patients’ outcomes when compared to traditional microscopic tools.

Objective

To investigate our own clinical and radiographic outcomes of ETSS for PA with its technical evolution over time as well as a significance of, having vs. lacking, the special endoscopic tools.

Methods

Included patients underwent ETSS for PA performed by the first author (AH). Prospectively recorded patients’ data concerning pre-, intra- and postoperative clinical and radiographic assessments were subject to analysis. The three groups of differently evolving ETSS techniques, beginning with mononostril (MN) to binostril ETSS with standard microsurgical instruments (BN1) and, lastly, binostril ETSS with specially-designed endoscopic tools (BN2), were examined for their impact on the intra- and, short- and long-term, postoperative results. Also, the survival after ETSS for PA, as defined by the need for reintervention in each technical group, was appraised.

Results

From January 2006 to 2012, there were 47, 101 and 72 ETSS, from 183 patients, in the MN, BN1 and BN2 cohorts, respectively. Significant preoperative findings were greater proportion of patients with prior surgery (p=0.01) and tumors with parasellar extension (p=0.02) in the binostril (BN1&2) than the MN group. Substantially shorter operative time and less amount of blood loss were evident as our technique had evolved (p<0.001). Despite higher incidence, and more advanced grades, of cerebrospinal fluid leakage in the binostril groups (p < 0.001), the requirement for post-ETSS surgical repair was less than the mononostril cohort (p=0.04). At six-month follow-up (n=214), quantitative radiographic outcome analysis was markedly superior in BN2. Consequently, long-term result was better in this latest technical group. Important negative risk factors, from multivariate Cox regression analysis, were prior surgery, Knosp grade, and firm tumor while BN1, BN2 and percentages of anteroposterior dimension PA removal had positive effect on longer survival.

Conclusion

The evolution of technique for ETSS for PA from MN to BN2 has shown its efficacy by improving intra- and postoperative outcomes in our study cohorts. Based on our results, not only that a neurosurgeon, wishing to start performing ETSS, should enroll in a formal fellowship training but he/she should also utilize advanced endoscopic tools, as we have proved its superior results in dealing with PA.

## Introduction

Transsphenoidal surgery (TSS) for pituitary adenoma (PA) has been performed in many centers around the globe for several decades with recent emergence of endoscopic TSS (ETSS). This novel technique has been shown to be as effective as, if not more than, the traditional transseptal microscopic TSS (mTSS) [1-11]. Therefore, it is not surprising to witness a recent shift from mTSS to ETSS [12]. Despite abundant publications for ETSS, few had studied comparative results between mono- vs. binostril access [13, 14]. Moreover, none had examined the effects of, having vs lacking, dedicated tools on patient’s outcomes. We report our own experience of ETSS with specific attention to the evolution of technique starting from mononostril to binostrils, both of which utilized standard microsurgical tools, and, finally, binostrils with advanced, endoscopic-designed, instruments. The primary objective was to investigate the effect, amongst different techniques, of the special equipment whether they resulted in dissimilar need-for-reintervention of PA. The secondary goal was to evaluate, both short- and long-term, clinical and radiographic outcomes of each distinctive technique of ETSS

## Materials and methods

The operating neurosurgeon (Ake Hansasuta, M.D.) has been particularly interested in pituitary surgery though he had no formal endoscopic endonasal skull base fellowship training. Transitioning from mTSS, after attending several didactic and cadaveric dissection courses, the first ETSS was performed at the Faculty of Medicine Ramathibodi Hospital in January 2006. Since the beginning of our ETSS, prospectively collected information from each case was recorded. The details of pre-, intra-, and postoperative data gathering and outcome evaluation are described below. With approval from the institutional review committee, retrospective analysis of the ETSS records was conducted. Patients who underwent ETSS, with confirmed pathology of pituitary adenoma or its apoplexy, performed by AH were included in this study. Aside from PAs with first-time ETSS, residual tumors were sorted into, based on their radiographic progression, with or without growth.

Preoperative and long-term postoperative assessments

Visual Function

With chief complaint of visual disturbance, each patient was examined for visual acuity (VA) by Snellen chart as well as visual field (VF) by automated Humphrey perimetry. After ETSS, visual function was evaluated using the same technique at six months. At this point, each patient’s postoperative visual (VA&VF) outcome was compared with preoperative value and was classified as improved, stable or worsened. For post-ETSS patients in need for urgent reoperation within 24 hours due to rapidly worsening vision, their ophthalmologic data were recorded at the time of reoperation. However, for patients with rapidly declining level of consciousness from postoperative apoplexy, without a possibility to obtain accurate visual function, their data were excluded from our ophthalmologic outcome assessment. Opthalmoplegia, if present before or after surgery, was documented.

Pituitary Hormone Function

After clinical suggestion of non-functioning or functioning PA, routine preoperative serum level of pituitary hormones including growth hormone (GH), age-specific insulin-like growth factor-1 (IGF-1), 8 AM cortisol, prolactin (PRL), thyroid stimulating hormone (TSH), free triiodothyronine (T3), free thyroxine (T4), testosterone, estradiol and gonadotropins, were obtained in every patient. Evidence of abnormally elevated hormone production before ETSS and standard criteria of functioning adenomas’ remission, i.e. GH [15], cortisol and ACTH [16], TSH [17] and PRL [18], were used for diagnosis and verification for postoperative hormonal cure. Diabetes insipidus (DI) after surgery was diagnosed by evidence of polyuria (urine output > 250 milliliter (mL) per hour for two consecutive hours), urine specific gravity < 1.005 and rising serum sodium over 145 milliequivalent per liter (mEq/L). Excluding patients with preexisting DI, new post-ETSS temporary DI was categorized by less than three months need for antidiuretic hormone supplement while long-term administration of it was considered permanent DI.

Radiographic Assessment

Preoperative imaging was obtained in all cases. Magnetic resonance imaging (MRI) was available for review in 92% of ETSS. On occasions, computed tomography (CT) scan was the only imaging modality before surgery (8%). Each patient’s sphenoid sinus was grouped into either sellar or presellar type. We did not perform ETSS on patients with concha type. In all cases, measured data of maximal diameter for transverse (X), anteroposterior (Y), and vertical (Z) aspects of their preoperative tumors were obtained. After 2008, volumetric measurement software became available allowing uniform calculation of the PA’s volume. If an MRI was obtained from an outside institute, it would be excluded from the postoperative volumetric analysis. Using Knosp et al., the tumor’s extension into parasellar region was graded [19]. Suprasellar extension was classified by the modified Hardy classification [20].

In case of no clinical indication for earlier radiographic study, such as postoperative apoplexy, the first MRI scan was obtained at six months after ETSS. Postoperative measurement of the tumors’ three dimensions (X, Y, Z) and volume were collected in similar fashion as before surgery. These pre- and postoperative dimensional and volumetric values were calculated for percentages of tumor removal (% removal) as below.

$percentages\, of\, tumor\, removal\, (dimension[X,Y\, or\, Z]\, or\, volume)\,=\tfrac{preoperative\, (X,Y,Z\, or\, volume)-postoperative\, (X,Y,Z\, or\, volume)}{preoperative\, (X,Y,Z\, or\, volume)}x100$

Patients without volumetric measurement either before or after ETSS were excluded from the % removal assessment by volume.

Evolution of surgical technique (Table 1)

The similarities in all groups were perioperative medication, i.e. routine hydrocortisone and antibiotic, and patient’s positioning using skull clamp with three-point rigid fixation. The left shoulder was elevated and supported to facilitate face turn toward the right side where operating surgeons stood. Either the right-sided abdomen or upper thigh was also prepped and draped for possible fat/fascia graft harvest. Antiseptic nasal wash followed by vasoconstrictive agent-soaked cottonoid packing in both nostrils was routinely done. After this point, each group of ETSS was different in its details of surgery.

First, mononostril ETSS technique (MN) was performed from January 2006 to May 2008 (29 months). Apart from utilizing an endoscope instead of microscope for visualization, most equipment was identical to mTSS. Many of them were bayonet neurosurgical instruments. For access, the right nostril was typically chosen unless this corridor was extremely narrow. After placement of a self-retaining nasal speculum, nasal mucosa below and medial to sphenoid ostium was coagulated away by monopolar cautery prior to sphenoidotomy. Contralateral access was gained by further extension of the bony and mucosal removal. Guided by cross-table lateral fluoroscopy, sellar floor was opened with osteotome and rongeur. After dura opening, using ring curettes of different angle and size, tumor resection began at its lower half followed by deeper portion. Subsequent resection of adenoma was carried out superiorly and, lastly, at its anterior part. If cerebrospinal (CSF) leakage was noted, depending on its grading by Eposito et al. [21], use of harvested fat and fascia graft was performed in every ETSS. In addition, for grade 2 and 3, every patient received lumbar drainage for CSF diversion.

In the second group of patients, the binostril ETSS technique utilizing standard mTSS tools (BN1) was employed from June 2008 to November 2010 (30 months). We abandoned the routine use of self-retaining nasal speculum entirely. With the same mTSS instruments, maneuvers for coagulation of nasal mucosa, sphenoidotomy, sellar enlargement and PA resection were unchanged from the MN technical group. Because of the availability of intraoperative stereotactic navigation, cross-table fluoroscopy usage had faded out. Additionally, flowable hemostatic agent was at hand to deal with troublesome hemorrhage. Unlike the mononostril group, if CSF leakage was observed, dura substitute and fibrin glue were applied for small leakage (grade 0 or 1) without lumbar drain placement. For larger defect (grade 2 or 3) or intraventricular entry, multi-layer closure by dura substitute, fat, fascia and fibrin glue were applied as well as lumbar drainage.

For the third and last technique, we performed binostrils ETSS with advanced endoscopic instruments (BN2). This modification of several steps took place from December 2010 until January 2012 (13 months).  First, preparation of mucosa for potential use of vascularized nasoseptal flap, instead of burning it away as in MN or BN1, was done in every case. Secondly, specially designed tools, for endoscopic use, were our new acquisitions. Examples of the equipment were such as bipolar cautery for endoscopic work, instead of regular ones, and transnasal low-speed drill, with different angle of cutting and diamond bur, that could reach sella turcica. The low-speed, 12,000 revolutions per minute (RPM), drill enabled ample bone opening. With the ability to remove bone of skull base, i.e. tuberculum sellae, planum sphenoidale or pterygoid plates, effectively, extended ETSS was, for the first time, possible whereas not applicable in the first two groups. Occasionally, a slim, with extra length, ultrasonic aspirator was of paramount when the neurosurgeon came across hard, or firm, consistency PA. These aforementioned tools helped vastly to overcome obstacle that hindered many problematic steps in the MN and BN1 groups. For sellar closure, in addition to multiple layer application as in BN1, vascularized nasoseptal mucosal flap, described by Hadad et al. [22], in high-grade CSF leakage, or entry into third ventricle was utilized. Lumbar drain usage was seldom.

**Table 1:** Details of different techniques in each endoscopic endonasal transsphenoidal surgery (ETSS) group for pituitary adenoma. CSF: cerebral spinal fluid

	Mononostril ETSS (MN)	Binostril ETSS with basic instruments (BN1)	Binostril ETSS with advanced instruments (BN2)
Period	January 2006-May 2008	June 2008-November 2010	December 2010-January 2012
Self-retaining nasal speculum	Yes	No	No
Sphenoid and septal mucosa	Monopolar cautery to burn away the mucosa	Monopolar cautery to burn away the mucosa	Preserved for possible vascularized nasoseptal mucosal flap
Sphenoid and sellar bone	Osteotome and rongeur	Osteotome and rongeur	Low-speed drill and rongeur
Intraoperative imaging guidance	Cross-table lateral view fluoroscopy	Cross-table lateral view fluoroscopy and, later, intraoperative stereotactic navigation	Intraoperative stereotactic navigation
Slim and long ultrasonic aspirator (for firm tumor)	No	No	Yes
Hemostasis	Monopolar, regular bipolar, gelatin sponge, oxidized cellulose polyanhydroglucuronic acid	Monopolar, regular bipolar, gelatin sponge, oxidized cellulose polyanhydroglucuronic acid, later, flowable hemostatic agent	As in BN1 plus specifically designed bipolar
CSF leakage (grade 0 or 1)	Fat, fascia	Dura substitute, fibrin glue	Dura substitute, fibrin glue
CSF leakage (grade 2 or 3)	Fat, fascia, lumbar drain	Fat, fascia, fibrin glue, lumbar drain	Fat, fascia, nasoseptal mucosal flap, seldomly lumbar drain

Intraoperative and short-term postoperative assessments

For every ETSS, operative time was charted from the start of nasal packing to the conclusion of surgery. The amount of blood loss, tumor consistency as well as surgical complication(s) were documented. After surgery, all patients were admitted to intermediate or intensive care unit for one night, or longer if necessary, with subsequent transfer to regular ward. Patients with low-grade (0 or 1) CSF leakage were kept flat in bed for two days whereas those with higher grades requiring three to four days due to lumbar drainage. The majority of patients in the BN2 group did not have CSF diversion but were kept in bed for the same length of time. Most patients did not have continued CSF leakage after this point. Without clinical evidence of CSF leakage, patients would subsequently be allowed to gradually increase the degree and the amount of time for upright position. If continued CSF leakage was observed during this period, the patient would remain recumbent with a lumbar drain, in the MN and BN1 groups, or without a lumbar drain in the BN2 group. Persistent CSF leakage, despite lumbar drainage, longer than one week prompted repeat TSS for repair whereas transcranial (TC) route was indicated for failure of cessation after the repeat TSS. Apart from persistent CSF leakage, patients with rapidly worsening visual function or level of consciousness from post-ETSS apoplexy would undergo emergency TSS or TC after radiographic confirmation.

Other than CSF leakage and apoplexy, our short-term postoperative complication included adverse events detected during the hospitalization and subsequent discharge within first 30 days after ETSS. Examples of the complications were epistaxis, temporary DI and serious infectious complication.

Follow-up and reintervention

After discharge, follow-up appointments with the neurosurgeon (AH) and endocrinologists were scheduled at two weeks, one and three months. At six months after ETSS, evaluation for visual function (VA&VF), endocrinologic and MRI studies were performed by the methods described previously. Also, the comparison between pre- and post-ETSS outcomes were assessed as mentioned earlier. Patients who underwent emergency re-surgery for post-ETSS apoplexy were excluded from the long-term assessments. If there was no requirement for reintervention at the first six-month postoperative visit, serial MRI would be obtained at six month intervals for the first two years and annually thereafter as long as there was no new therapy.

For patients with residual tumor, decision for reintervention was based on various factors as followed. Frequent rationales for reoperation, ETSS or TC, were persistently high level of hormones in functioning adenomas, symptomatic compression of surrounding structure and recurrent growth of residual tumor. Patient’s age, co-morbidities and, in some, preference were also taken into consideration for reoperation. Apart from surgery, patients were given information in regard to their options for non-surgical treatments such as radiotherapy or medical treatment. Some patients elected to watch their residual tumors by periodic MRI surveillance. After reintervention, the patient would be censored from further data record. For those elected to undergo another ETSS, each patient would start as a new case, who had prior surgery, in the same or different ETSS group.

Data analysis

Utilizing Stata software version 12.0 (Stata Corp., College Station, TX), pre-, intra- and postoperative parameters together with outcomes comparison amongst stratified MN, BN1 and BN2 groups were executed with unpaired t-test, rank tests, chi-square and Fisher’s exact test as appropriate, assuming statistical independence. To identify risk factors for reintervention, Cox proportional hazard regression models were used for both univariate and multivariate analyses. Kaplan-Meier graphs were computed, arranged by the three technical groups, to assess the reintervention-free survival. P-value of <0.05 was considered statistically significant.

## Results

Patient demographics (Table 2)

**Table 2:** Preoperative demographic, clinical, visual, endocrine presentation and radiographic features of patients undergoing endoscopic transsphenoidal surgery (ETSS) from January 2006 to 2012 (cm = centimeter, mL = milliliter)

Patient demographics	Mononostril ETSS (MN)	Binostril ETSS with basic instruments (BN1)	Binostril ETSS with advanced instruments (BN2)	p-value
Number of patients	43	89	51	
Number of operations	47	101	72	
Male: no.(%)	19(44)	38(43)	20(39)	0.865
Age(year): mean(SD)	49.7(10.7)	50.5(12.6)	50.1(13.6)	0.937
Duration of symptom(month): median(range)	18(0.003-84)	20.5(0.006-72)	24(0.06-84)	0.156
Abnormal visual acuity: no.(%)	32(68)	67(66)	39(54)	0.183
Abnormal visual field: no.(%)	33(70)	66(65)	38(53)	0.149
Non-functioning adenoma: no.(%)	40(85)	76(75)	52(72)	0.254
Functioning adenoma: no.(%)	7(15)	25(25)	20(26)	
- Prolactinoma: no.(%)	0	2(2)	0	
- Growth hormone: no.(%)	5(11)	20(19)	15(21)	
- Adrenocorticotropic hormone: no.(%)	1(2)	2(2)	3(4)	
- Thyroid stimulating hormone: no.(%)	0	1(1)	1(1)	
Apoplexy presentation: no.(%)	1(2)	4(4)	2(3)	0.752
- Altered level of consciousness: no.(%)	1(2)	2(2)	2(3)	
- Opthalmoplegia: no.(%)	1(2)	3(3)	2(3)	
Prior surgery: no.(%)	8(17)	40(40)	29(40)	0.01
No. of prior surgery: no.(%)				
0	39(83)	61(60)	43(60)	0.01
1	6(13)	32(32)	15(21)	
2	2(4)	8(8)	12(17)	
3	0	0	2(3)	
Residual tumor with growth: no.(%)	5(10)	26(26)	13(18)	0.17
Residual tumor without growth: no.(%)	3(6)	14(14)	16(22)	0.08
Prior radiation: no.(%)	1(2)	4(4)	3(4)	0.88
Preoperative radiographic features				
Presellar type: no.(%)	2(4)	14(14)	12(17)	0.125
Macroadenoma: no.(%)	44(94)	98(97)	69(96)	0.621
Preoperative tumor measurements				
- Transverse diameter(cm): mean(SD)	2.2(0.9)	2.4(0.9)	2.3(0.8)	0.395
- Anteroposterior diameter(cm): mean(SD)	2.2(0.8)	2.4(0.9)	2.2(0.8)	0.39
- Vertical diameter(cm): mean(SD)	2.3(1.1)	2.6(1.2)	2.5(1.1)	0.692
- Tumor volume(mL): mean(SD)	N/A	12.1(10.2) n=72	11.9(9.0) n=60	0.988
Parasellar extension ( Knosp Grade)				0.02
- Grade 0-2: no.(%)	34(72)	48(48)	39(54)	
- Grade 3-4: no.(%)	13(28)	53(52)	33(46)	
Suprasellar extension (Modified Hardy grade)				0.958
- Grade 0, A and B: no.(%)	28(60)	58(57)	41(57)	
- Grade C: no.(%)	19(40)	43(43)	31(43)	

Between January 2006 and January 2012, two hundreds and fifty-eight consecutive ETSS have been performed at the Faculty of Medicine Ramathibodi Hospital by the, first author, neurosurgeon (AH). Thirty-eight non-PA, such as craniopharyngioma, meningioma, arachnoid cyst, Rathke’s cleft cyst, epidermoid cyst, chordoma, and non-diagnostic specimens, were excluded, leaving 220 operations for review. Among them, 47, 101 and 72 surgeries were in the MN, BN1 and BN2 group, respectively. There were 21 patients who underwent two ETSS and two patients with three ETSS. Cases with prior surgery in the binostril cohorts (40%) were more frequent than in the mononostril group (17%)(p=0.01). Preoperative radiographic studies amongst the cohorts revealed no significant dimensional or volume measurement difference. Nevertheless, higher Knosp grade (3&4) was more frequent in the BN1&2 groups than MN (p=0.02).

Intraoperative findings and short-term outcomes (Table 3)

**Table 3:** Intraoperative and short-term (30-day) postoperative results of patients undergoing endoscopic transsphenoidal surgery (ETSS) from January 2006 to 2012 (mL = milliliter, TSS = transsphenoidal, TC = transcranial)

	Mononostril ETSS (MN)	Binostril ETSS with basic instruments (BN1)	Binostril ETSS with advanced instruments (BN2)	p-value
Operations: no.	47	101	72	
Intraoperative findings and complications				
Operative time(hour): mean(SD)	5.1(1.2)	4.4(1.3)	3.6(0.9)	<0.001
Estimated blood loss(mL): median(range)	420(170-1,200)	295(80-1500)	250(70-580)	<0.001
Excessive venous bleeding causing premature termination of ETSS: no.(%)	3(6)	0	0	0.004
Firm tumor: no.(%)	1(2)	11(11)	12(17)	0.045
Intraoperative CSF leakage: no.(%)	13(28)	54(54)	46(64)	<0.001
CSF leakage grade				<0.001
- 0:no.(%)	34(72)	47(46)	26(36)	
- 1:no.(%)	7(15)	18(18)	9(12)	
- 2:no.(%)	6(13)	30(30)	23(32)	
- 3:no.(%)	0	6(6)	14(20)	
Carotid artery injury: no.(%)	0	1(1)	0	0.553
Postoperative complications (within 30 days)				
Death:no.(%)	0	1(1)	0	0.553
Postoperative persistent CSF rhinorrhea requiring surgery either TSS or TC: no.(%)	3(21)	4(7)	1(2)	0.04
Temporary diabetes insipidus: no.(%)	4(8)	9(9)	7(10)	0.912
Postoperative apoplexy: no.(%)	0	3(3)	0	0.167
Worsening visual function: no.(%)	0	1(1)	0	0.567
Meningitis: no.(%)	0	1(1)	1(1)	0.733
Septicemia: no.(%)	0	2(2)	0	0.305
Epistaxis: no.(%)	1(2)	2(2)	1(1)	0.944

Significantly shorter operative time and less amount of blood loss, as our ETSS technique had evolved, was remarkable (p<0.001). Compared to no occurrence in the binostril groups, three surgeries (6%) from the MN group came to premature end due to uncontrollable venous bleeding (p=0.004). Firm tumors were more commonly encountered in the latter groups than MN (p=0.045) which could be related to higher incidence of prior surgery in the binostril groups as described earlier. In spite of greater incidence and advanced grades of CSF leakage observed during ETSS in the binostril, especially the BN2, groups (p<0.001), postoperative persistent leakage requiring additional surgery, either TSS or TC, for repair was substantially less (p=0.04). The rest of intraoperative and short-term, 30-day, analyses were not different among groups. There were one carotid artery injury and one death in the BN1 group. The carotid artery event occurred while rongeuring off sellar bone, albeit real-time navigation. With successful intraoperative hemostasis, the patient did not suffer stroke after an endovascular procedure and lived normal life thereafter. One death occurred in another patient who had previously undergone multiple surgeries, both TC and mTSS, and radiotherapy. This patient had recurrent growth of the residual tumor resulting in progressive optic pathway compression. Unable to resect much tumor due to its firm consistency, apoplectic event, causing rapid decline in mental status ensued. Despite successful evacuation of the hemorrhagic transformation and CSF leak repair via TC approach, he, later, deteriorated from fulminant meningitis/ventriculitis. The patient passed away even with broad-spectrum intravenous antibiotics and aggressive resuscitation. Two additional patients, in the BN1 cohort, suffered postoperative apoplexy with declining level of consciousness in one and significantly worsening vision in the other. Both survived after emergency evacuation of hemorrhagic transformation of suprasellar residual tumors via TC. There was no new postoperative ophthalmoplegia from all technical cohorts.

Long-term outcomes (Table 4)

**Table 4:** Long-term follow-up of patients (n=214) undergoing endoscopic transsphenoidal surgery(ETSS) from January 2006 to 2012

	Mononostril ETSS (MN)	Binostril ETSS with basic instruments (BN1)	Binostril ETSS with advanced instruments (BN2)	p-value
Operations: no.	47	101	72	
Lost follow-up: no.(%)	1(2)	3(3)	2(3)	0.957
Follow up time(month): median(range)	96(6-120)	72(6-96)	55(6-69)	<0.001
Reintervention: no.(%)	37(80)	71(72)	40(57)	0.02
Functional adenoma cured: no.(%)	3/7(43)	10/24(42)	7/18(38)	0.916
Permanent diabetes insipidus: no.(%)	0	1(1)	1(1)	0.745
Available subjects for postoperative visual assessment	n = 32	n = 63	n = 39	
Visual acuity improved: no.(%)	28(88)	58(92)	38(97)	0.279
Visual acuity worsen: no.(%)	0	1(2)	0	0.567
Visual field improved: no.(%)	29(90)	56(90)	36(94)	0.719
Available subjects for pre- and postoperative three dimensional measurements	n = 46	n = 98	n = 70	
Percentages of tumor removal (transverse): median(range)	29(0-100)	36(0-100)	64(0-100)	<0.001
Percentages tumor removal (anteroposterior): median(range)	40(0-100)	39(0-100)	62(5-100)	<0.001
Percentages of tumor removal (vertical): median(range)	50(0-100)	46(0-100)	69(17-100)	<0.001
Available subjects for pre- and postoperative tumor volumetric calculation	N/A	n = 66	n = 54	
Percentages of tumor removal (volume): median(range)	N/A	75(5-100)	84(50-100)	0.04
Tumor removal > 80% volume: no(%)	N/A	39(59)	41(75)	0.01

With three patients lost to follow-up and other three patients with postoperative apoplexy, 214 cases were available for long-term assessment. The incidence of temporary and permanent DI was not significantly different among the three technical groups. At the first six-month post-ETSS visit, the % removal clearly demonstrated, by X-Y-Z dimensions. Superior PA resection in the BN2 over BN1 and the BN1 over MN group was obvious (p<0.001). Within the binostril groups, from 120 subjects with available volumetric calculation, the BN2 showed better % removal over the BN1 (p=0.04). In addition, the number of cases with > 80% tumor removal was greater in BN2 than BN1 group (p=0.01).

**Figure 1:**
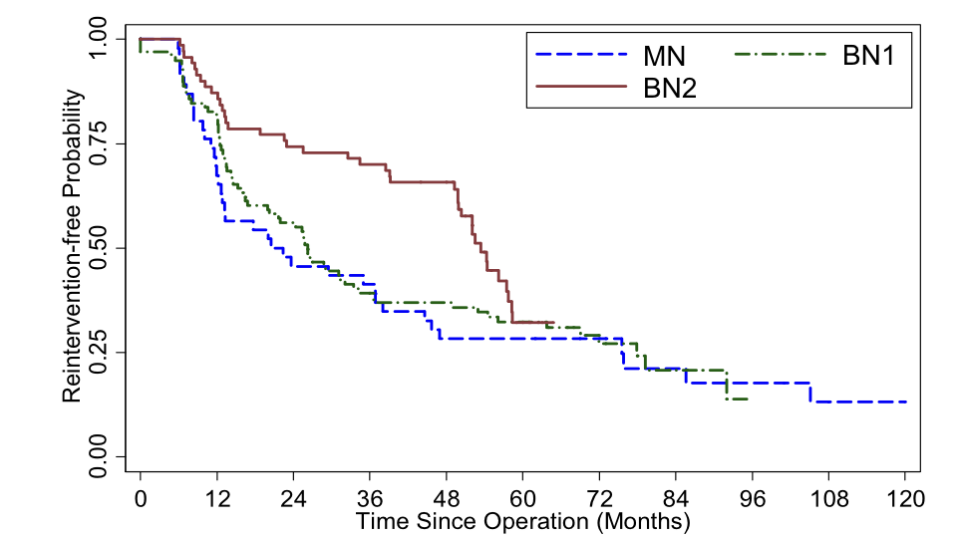
Kaplan-Meier survival curve illustrates reintervention-free probability, by operative group, from all patients who underwent endoscopic transsphenoidal surgery(ETSS) for pituitary adenoma at Ramathibodi hospital, with available follow-up (n = 214). Log-rank test p-value = 0.066 (MN = mononostril ETSS, BN1 = binostril ETSS with basic instruments and BN2 = binostril ETSS with advanced instruments)

Reintervention-free interval in the BN2, compared with BN1 and MN, group, from Kaplan-Meier survival curve, almost reached statistical significance (p=0.066) for longer reintervention-free duration of BN2 when all follow-up cases were included(Figure 1).

**Figure 2:**
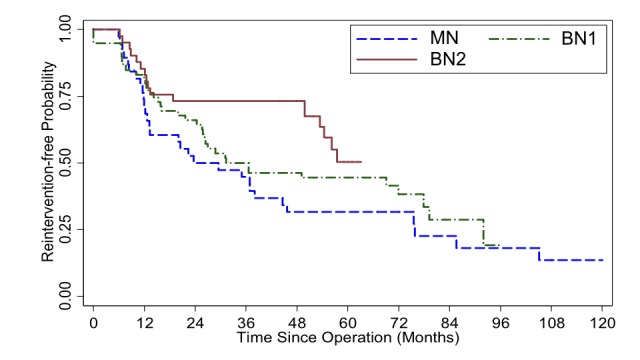
Kaplan-Meier survival curve illustrates reintervention-free probability, by operative group, from patients who underwent their first endoscopic transsphenoidal surgery (ETSS), also first surgery, for pituitary adenoma at Ramathibodi hospital, with available follow-up (n = 138). Log-rank test p-value = 0.077 (MN = mononostril ETSS, BN1 = binostril ETSS with basic instruments and BN2 = binostril ETSS with advanced instruments)

When only first surgery (ETSS) performed at our institute, excluding those with prior TSS or TC, were considered (n=138), the curve also displayed a trend (p=0.077) with BN1 separating itself further from MN, more than the first, all-patient-included, graph (Figure 2).

**Figure 3:**
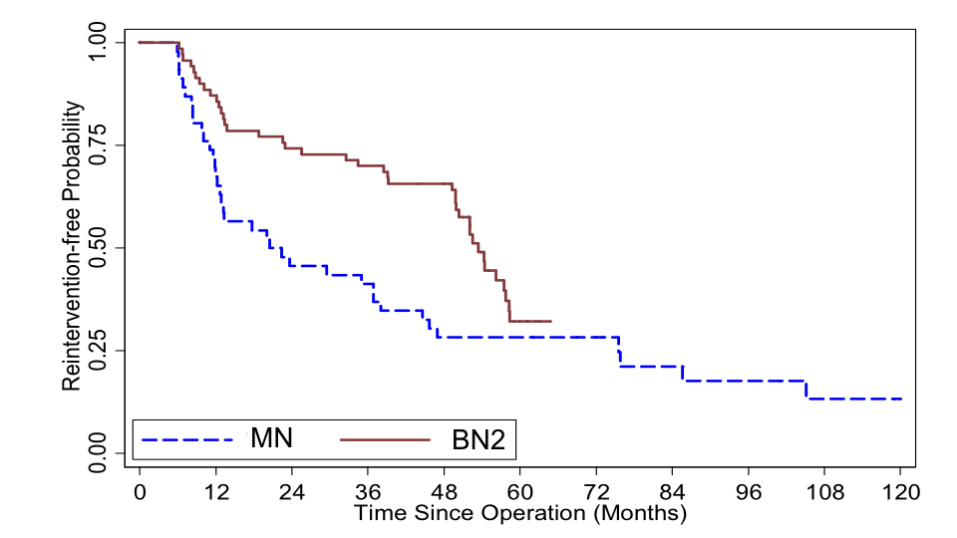
Kaplan-Meier survival curve illustrates reintervention-free probability comparing mononostril endoscopic transsphenoidal surgery (ETSS) [MN] and binostril ETSS utilizing advanced instruments [BN2] from all patients who underwent ETSS for pituitary adenoma at Ramathibodi hospital (n = 116). Log-rank test p-value = 0.030

**Figure 4:**
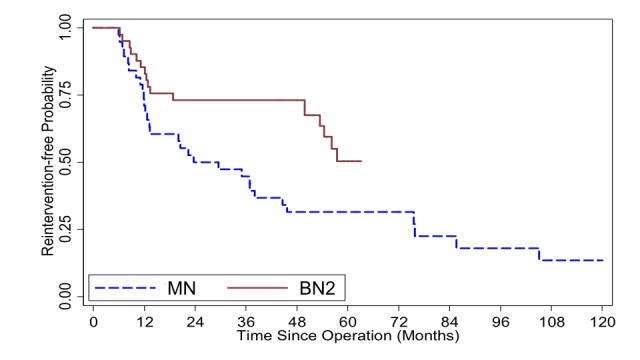
Kaplan-Meier survival curve illustrates reintervention-free probability comparing mononostril endoscopic transsphenoidal surgery (ETSS) [MN] and binostril ETSS utilizing advanced instruments [BN2] from patients who underwent their first ETSS, also first surgery, for pituitary adenoma at Ramathibodi hospital (n = 79). Log-rank test p-value = 0.017

Upon direct comparison between BN2 and MN group, significant survival, in all cases (p=0.03) and in first-time ETSS (p=0.017), was perceived (Figures 3, 4).

Despite superior radiographic outcomes after binostril ETSS over MN, from 134 cases with preoperative visual complaints, there was only a trend for slightly better visual function. This improvement, however, did not reach statistical significance (p=0.279).

Analyses of risk factors for reintervention

Univariate analyses of various risk factors for reintervention revealed that the BN2 group and apoplexy presentation were associated with favorable outcomes. In contrast, prior surgery and its increasing numbers of procedures had adverse impact on survival-free period. Other negative factors were sphenoid sinus with presellar type, Knosp grade and firm tumor. While the preoperative transverse (X) and anteroposterior (Y) dimensions were correlated with poorer outcome but, to our wonder, not the vertical (Z) measurement. Nevertheless, the postoperative % removal by dimensional and volumetric measurements linked with longer reintervention-free duration (Table 5).

**Table 5:** Cox proportional hazards regression models showing pre-, intra- and postoperative risk factors for reintervention (n=214), univariable analysis *= adjusted for clustering on patients (cm = centimeter, mL = milliliter)

Factors	Hazard ratio (95%CI*)	p-value*
Endoscopic transsphenoidal surgery (ETSS) technique		
Mononostril ETSS (MN) [reference]	1	N/A
Binostril ETSS with basic instruments (BN1)	0.92(0.59-1.44)	0.711
Binostril ETSS with advanced instruments (BN2)	0.62(0.38-1.01)	0.046
Preoperative risk factor		
Age (per year increase)	0.99(0.98-1.01)	0.855
Male vs Female	0.99(0.72-1.37)	0.966
Visual presentation	1.15(0.80-1.63)	0.451
Apoplexy presentation	0.32(0.11-0.89)	0.028
Asymptomatic presentation	1.18(0.76-1.82)	0.459
Prior surgery	1.78(1.30-2.44)	<0.001
Number of prior surgery (per number of surgery increase)	1.37(1.11-1.68)	0.003
Preoperative transverse diameter (per cm increase)	1.29(1.05-1.59)	0.015
Preoperative anteroposterior diameter (per cm increase)	1.08(1.07-1.64)	0.010
Preoperative vertical diameter (per cm increase)	1.08(0.93-1.25)	0.306
Preoperative tumor volume (per mL increase)	1.00(0.98-1.03)	0.76
Presellar type	1.47(1.01-2.15)	0.044
Macroadenoma	1.78(0.56-5.67)	0.326
Hardy grade (per grade increase )	1.13(0.95-1.34)	0.167
Knosp grade (per grade increase)	1.81(1.45-2.25)	<0.001
Duration of symptoms (per month increase)	81.4(71.4-91.3)	0.57
Intraoperative risk factor		
Firm tumor	1.70(1.01-2.84)	0.044
CSF leakage (per grade increase)	0.95(0.82-1.11)	0.541
Operative time (per hour increase)	1.08(0.94-1.24)	0.277
Postoperative risk factor		
Visual symptom improvement	1.02(0.73-1.42)	0.928
Pathology		
Non-functioning adenoma [reference]	1	N/A
Growth hormone-producing adenoma	1.23(0.73-2.06)	0.439
Other functioning adenoma	0.60(0.29-1.27)	0.182
Extent of tumor removal		
Percentages of tumor removal (transverse) (per % removal)	0.978(0.972-0.985)	<0.001
Percentages of tumor removal (anteroposterior) (per % removal)	0.975(0.969-0.982)	<0.001
Percentages of tumor removal (vertical) (per % removal)	0.978(0.971-0.985)	<0.001
Percentages of tumor removal (volume) (per % removal; n=120)	0.980(0.968-0.992)	<0.001

Further statistical examination, by multivariate analyses, reconfirmed the positive effect of the BN2. Additionally, it also disclosed increased survival power of the BN1 over MN. Prior surgery, Knosp grade and firm tumor were reiterated as being negative factors. Interestingly, the % removal of anteroposterior (Y), not the transverse (X) or vertical (Z), axis also correlated with longer reintervention-free time (Table 6).

**Table 6:** Multivariable Cox regression analysis of risk factors for reintervention (n=214) *= adjusted for clustering on patients

Risk factor	Hazard ratio (95%CI*)	p-value*
Mononostril ETSS (MN) [reference]	1	N/A
Binostril ETSS with basic instruments (BN1)	0.57(0.36-0.89)	0.013
Binostril ETSS with advanced instruments (BN2)	0.34(0.21-0.56)	<0.001
Prior surgery	1.68(1.28-2.39)	0.004
Knosp grade (per grade increase)	1.84(1.48-2.29)	<0.001
Firm tumor	1.67(1.03-2.71)	0.036
Percentages of tumor removal (anteroposterior) (per % removal)	0.98(0.97-0.99)	<0.001

## Discussion

Endoscopic endonasal skull base surgery, with clear advantages of minimal brain manipulation and superb panoramic view visualization, is here to stay. Published literature reported outcomes of PA surgery comparing between mTSS vs. ETSS with a trend for superior result towards the endoscopic method especially PA with suprasellar extension [1, 4-6, 9]. In addition, mono- vs binostril access had been reviewed and reports of binostril ETSS with slightly better results were described [13, 14]. Considering abundant information with regard to various techniques of ETSS, none had yet explored the influence of, having vs. lacking, sophisticated endoscopic tools on clinical and radiographic outcomes. Therefore, this study aimed to find out if there was relevant impact from advanced endoscopic equipment over standard mTSS tools in pure ETSS for PA.

Similar to previous publications [23, 24], it is undeniable that one immensely influencing factor in this study must have been our own learning curve acquired over time. Its effect was apparent by shorter operative time and less amount of blood loss as the ETSS had evolved. Without a formal fellowship, the neurosurgeon (AH) had gone through a very steep learning curve struggling with a narrow corridor, during MN, to a wider passage, with BN1, both of which without dedicated instrument. Finally, larger opening with more defined tools in the BN2 group facilitated desirable outcomes. Hence, in addition to adequate training, utilization of advanced instruments is of paramount per our clinical and radiographic results. Although the majority of clinical outcomes were not different between MN and BN1 group, greater extent of tumor resection, as shown by three-dimensional (X, Y, Z) % removal, was noted in the binostril access. Regardless of similarity of the survival curve in Kaplan-Meier estimator, there was positive effect on the reintervention-free duration, by multivariate analysis, of the BN1 over the MN group (p=0.013) indicating some impact of binostril technique (Table 6). This is, perhaps, the true logic that larger corridor, together with wider viewing angle, ameliorating deeper reach, evidently by the frequency and higher grades of CSF leakage in the bi- over mononostril approach. After acquisition of advanced tools, even better exposure, with expanded TSS i.e. transtuberculum or transpterygoid, and tumor resection enabled superior outcomes in the BN2 group.

Intraoperatively, firm tumors in the binostril more than the mononostril group were frequently observed. We believed this phenomenon could partly be from greater numbers of cases with prior surgery, thus, more fibrosis within PAs. Ineffective removal of firm tumors by ring curette in MN and BN1 group was overcome by utilizing long and slim ultrasonic aspirator in BN2. Not only more aggressive PA resection in the binostril groups brought about no, or smaller, residual tumor and, consequently, longer survival but also larger defect of CSF leakage. However, with modification of sellar closure method, the need for additional surgical repair for persistent post-ETSS CSF rhinorrhea was, instead, significantly lower. This is, most likely, owing to vascularized nasoseptal mucosal flap utilized in BN2 cohort despite seldom use of lumbar drainage.

Another apparent difficulty in our early ETSS was managing profuse hemorrhage. It was an extreme challenge dealing with intercavernous sinus, or cavernous sinus itself, outpouring via mononostril approach. Controlling high-pressure venous bleeding, more than oozing, typically required at least two, preferably three, hands to accomplish the task. To no wonder, three ETSS in the MN group had to be prematurely aborted due to failure of adequate hemostasis to proceed with the next step of surgery. In contrast, with binostril access yielding wider exposure, hence, better maneuverability, along with handy flowable hemostasis, no ETSS had to be terminated due to the same reason. Although wider viewing angle was provided by the BN1 technique, a lack of specially designed tools could have affected unforgiving complication(s) namely the postoperative apoplexy from huge bulk of remaining PA at the suprasellar space. While the incidence did not reach statistical significance (p=0.167), by having sophisticated equipment, there was no postoperative apoplexy in the BN2 group, probably by maximizing access and ability to reach deeper, consequently, smaller or no residual tumor, than the BN1 group. In addition, much as bayonet neurosurgical instruments allow surgeon to work without the operating hands obstructing the line of sight via microscope for mTSS, this extra angle consumes more space during endoscopic work creating chopstick effect hindering maneuverability. Utilizing these mTSS tools, that was rather cumbersome, could have been one of other influencing factors resulting in less desirable outcomes in MN and BN1 compared to BN2.

At six-month follow up, it was somewhat disappointing that post-ETSS visual improvement did not reach statistical difference in light of obviously better % tumor removal as this technique had evolved. On the opposite, lacking equipment to tackle firm PA, one worsened visual function from postoperative apoplexy occurred in the BN1 group. Although this did not reach statistical significance, no patient in the BN2 cohort suffered such complication when advanced instruments were available. Despite only a trend for longer survival in BN2 by reintervention-free Kaplan-Meier curves (Figure 3), this inclination became more evident when cases with prior surgery were excluded. Further separation of the curves between BN1 and MN was recognized (Figure 4). Logical explanation for this should be the higher % removal in BN2 over BN1, by advanced tools, and BN1 over MN, by larger exposure, that more aggressive PA resection left smaller residual tumor behind. Thus, PA recurrence was delayed. When ETSS techniques were not considered, as in multivariate analysis, risk factors, for worse outcome, were similar to prior literatures, such as prior surgery, Knosp grade and firm tumor [25-27]. Interestingly, besides binostril approaches, another positive factor was the % tumor removal in the anteroposterior dimension. One probable explanation could be the fact that higher proportion of, or total, PA resection obliged reaching deeper (anteroposterior dimension) into the sella and the tumor. This could, in part, coincide with the increased frequency and higher grades of CSF leakage in the bi- over the mononostril group.

Potential drawbacks in this study are as followed. We admit that there certainly existed selection bias for patients undergoing ETSS in mono- vs binostril group. At the beginning of the first cohort (MN), during the transition from mTSS to ETSS, the neurosurgeon (AH) still performed mTSS in selected functioning PAs for better maneuverability hoping for higher chance of cure. If these cases were to undergo mononostril ETSS, its result could have probably been poorer. In contrast, no patient underwent mTSS in during the binostril ETSS period. One other example of significant selection bias was that, in the MN group, we elected not to perform ETSS for PA with intraventricular extension due to the lack of experience. Furthermore, for fear of major complication as much as awareness of limited maneuverability via undersized corridor, these patients in the mononostril group underwent TC instead of ETSS and then became subjects in the binostril approaches for residual tumor afterwards. This could potentially be the important reason why there was no postoperative apoplexy in MN group. After acquiring more experience along with wider access gained by the binostril method, profiting less cumbersome chopstick effect, cases with this level of difficulty (PA with intraventricular extension) underwent ETSS in the BN1 (9%) and BN2 (11%) group. It might be a valid explanation why three patients suffered postoperative apoplexy in the BN1 cohort. Later, with availability of advanced, slim and long, instruments in the BN2 group, allowing ample exposure and, as a consequence, greater extent of PA removal, thus, there was no postoperative apoplexy as earlier discussed. The other selection bias was evident in the higher proportion of cases with previous surgery, presellar type and parasellar extension, in the BN1&2 group. This particular preference was, again, driven by more experience and advanced tools availability.

Apart from those above-mentioned selection bias, our second pitfall could potentially be from some discrepancy between measurements of, and comparison between, different radiographic modalities. Although the preoperative imaging measurements were mostly from MRI, a few cases had only CT scan before ETSS (8%), making comparisons between different pre- vs postoperative dimension (X, Y, Z) data somewhat less accurate. These cases, however, were omitted from the volumetric calculation. While measuring X, Y, Z provided some objective data, nonetheless, not all PA were perfectly circular or elliptical. There were invasive tumors with odd configurations. Therefore, measuring the widest dimensions could not be universally informative. The other drawback is the different criteria for ETSS failure. We used the definition of “reintervention-free” instead of “progression-free” survival unlike many of the previous reports. Some patients, particularly in the BN2 group, underwent additional treatment before tumor exhibited growth. Reintervention was decided even without clinical or radiographic progression (Table 2). As a consequence, based on the Kaplan-Meier survival estimator, one may notice that, at 48-60 months after ETSS, the BN2 cohort’s curve dropped to almost at the same level as MN or BN1 whereas, up to its first 48 months, it was distinctively superior than the basic-instrument cohort. It appeared as though utilizing advanced tools did not yield long-term advantage (Figures 1-4). This was also an occurrence from our recent selection bias, again, for further treatment in asymptomatic patients most of whom had non-functioning PAs. Some of them underwent redo ETSS in the BN2 group despite no or minimal growth. This particular tendency was due to recent acquisition of more advanced equipment i.e. intraoperative MRI [28], transnasal highspeed drill, bipolar sealer, slim&long doppler ultrasound. Owing to the awareness of, given enough time, residual PA could eventually regrow [29, 30]. Thus, with even better equipment as mentioned, we were inclined to offer, preferably young and healthy, patients repeat ETSS. On the contrary, similar patients in the previous (MN and BN1) groups would have just deferred surgical treatment but continued radiographic surveillance until PA growth was perceived.

In spite of the aforementioned selection bias and more negatively-influencing pre- and intraoperative parameters, the binostril ETSS cohorts demonstrated better clinical and radiographic aftermaths. The dedicated tools were pivotal pieces of success in the BN2 group enabling, near-complete or, complete resection, therefore, resulting in desirable short- and long-term outcomes. This evidence-based data put an emphasis on how modern day ETSS for PA should be performed particularly in those with complex tumor and sphenoid sinus anatomy. Nevertheless, the meaningful comparison between mono- vs binostril ETSS ought to be fairly evaluated by randomized controlled trial utilizing same sophisticated instruments by the same, highly experienced, surgeon. Although a prospectively designed study, to explore within ETSS comparing outcomes between with vs. without special equipment, would ideally generate improved class of evidence for this matter, it would be rather unethical considering previous publication’s and our current results.

Lastly, while this research may not alter the practice of neurosurgeons already utilizing advanced tools, our information ought to be somewhat invaluable for those working in under-developed or developing countries with limited budgets. Dedicated endoscopic equipment and disposable items can immensely escalate the overall surgical care cost of patients with PA when compared to mTSS or ETSS using mTSS tools. For a neurosurgeon with interest in ETSS but lacking formal fellowship and advanced endoscopic instrument training, mitigating major complication(s) by selecting good surgical candidates, i.e. simple PA with straightforward sphenoid sinus anatomy, for his/her start of ETSS is strongly advised.

## Conclusions

Success of ETSS for PA demands several factors. It requires not only tremendous surgical skill and experience but also specially-designed instruments in order to achieve the desirable results. This study demonstrated significantly superior intra- and postoperative clinical and radiographic outcomes in the advanced- over the basic-instrument group as well as the bi- over the mononostril group. Any neurosurgeon willing to commence ETSS, particularly without a fellowship training, ought to be fully furnished with advanced tools. Prior surgery, higher Knops grade and firm tumor were, again, associated with poorer outcome of ETSS for PA.
